# Biotransformation of Sumatriptan by *Staphylococcus aureus*, *Bacillus subtilis*, *Pseudomonas aeruginosa* and *Salmonella enterica* subsp. *enterica*

**DOI:** 10.3390/molecules29174226

**Published:** 2024-09-06

**Authors:** Muhammad Jehangir, Mohammad Saeed Iqbal, Usman Aftab

**Affiliations:** 1 Department of Chemistry, Forman Christian College, Lahore 54600, Pakistan; m.jehangir@novamed.com.pk; 2 Department of Pharmacology, University of Health Sciences, Khayaban-e-Jamia Punjab, Lahore 54600, Pakistan; usmanaftab@uhs.edu.pk

**Keywords:** bacterial transformation, triptan, LC-MS, metabolism, biodegradation

## Abstract

This study aimed at the biotransformation of sumatriptan by *Staphylococcus aureus*, *Bacillus subtilis*, *Pseudomonas aeruginosa* and *Salmonella enterica* subsp. *enterica* and the identification of the drug metabolites by liquid chromatography–mass spectrometry. The drug was incubated with the organisms in tryptic soya broth at 37 °C. The broth was filtered and subjected to liquid chromatography–mass spectrometry. The metabolites identified by the use of mass spectral (+ve ion mode) fragmentation patterns were (3-methylphenyl)methanethiol (*Bacillus subtilis*), 1-(4-amino-3-ethylphenyl)-N-methylmethanesulfonamide (*Salmonella enterica* subsp. *enterica*) and 1-{4-amino-3-[(1E)-3-(dimethylamino)prop-1-en-1-yl]phenyl}methanesulfinamide (*Salmonella enterica subsp. enterica*, *Bacillus subtilis*, *Pseudomonas aeruginosa*, *Staphylococcus aureus*). These metabolites exhibit high gastrointestinal absorption, no blood–brain barrier permeability (except (3-methylphenyl)methanethiol), a bioavailability score of 0.55 and no inhibitory effect on CYP2C19, CYP2C9, CYP2D6, CYP3A4 or cytochrome P450 1A2 (except (3-methylphenyl)methanethiol), as determined by SwissADME software ver. 2024. The metabolites appear to be more toxic than the parent drug, as suggested by their calculated median lethal dose values. All four organisms under investigation transformed sumatriptan to different chemical substances that were more toxic than the parent drug.

## 1. Introduction

The biotransformation of drug substances is an important research area with regard to drug metabolism as it can influence the efficacy and safety of a drug after its chemical alteration by living organisms. Biotransformation can also result in the selective synthesis of new medicinal compounds [1]. These transformations provide economical and environmentally friendly synthesis methods. Drug metabolism may result in the biotransformation of drug molecules to pharmacologically active or inactive metabolites, which may be toxic or non-toxic. This knowledge determines the safety and efficacy of drug substances. The drug substances that are resistant to metabolism tend to be more effective for longer periods of time than those that are rapidly metabolized. Biotransformation has been successfully used to produce new molecules, especially those that are usually synthesized through multistep processes or sometimes impossible to synthesize by routine methods [2]. Several microorganisms have been employed for the production of useful quantities of metabolites [3] while avoiding the use of toxic reagents and complex reaction conditions. Biotransformations by organisms usually involve oxidation, reduction, hydrolysis, isomerization, condensation and the introduction of new functional groups [2].

The present work presents the results of the biotransformation of sumatriptan by *Staphylococcus aureus* (*S. aureus*), *Bacillus subtilis* (*B. subtilis*), *Pseudomonas aeruginosa* (*P. aeruginosa*) and *Salmonella enterica* subsp. *enterica* (*Salmonella* spp.). A careful survey of the literature indicated that no such study has been reported to date. Sumatriptan, 1-{3-[2-(dimethylamino)ethyl]-1H-indol-5-yl}-N-methylmethanesulfonamide (**I**), is a widely used drug in the treatment of migraine and cluster headaches [4,5,6]. It is administered orally, intranasally, or by subcutaneous injection [6]. Sumatriptan is rapidly but incompletely absorbed when given orally and undergoes first-pass metabolism, resulting in low absolute bioavailability. The therapeutic effects generally occur within three hours. Primarily, it is a serotonin 5-HT1B/1D receptor agonist [7], associated with side effects including fatigue, tingling, vomiting, chest pressure, serotonin syndrome, heart attacks, strokes, seizures and vertigo [6]. Its mechanism of action and safety during pregnancy and breastfeeding are unknown [8]; these could be explained if the metabolic pathway of the drug is identified. It is reported to be metabolized by oxidative deamination of its dimethylaminoethyl residue by monoamine oxidase A (MAO A) and not by cytochrome P450 (CYP)-mediated demethylation [9]. A prior study reported two metabolites, N-desmethyl sumatriptan and N,N-didesmethyl sumatriptan, generated by CYP1A2, CYP2C19 and CYP2D6. Thus, there are clinical challenges regarding the administration of the drug. 
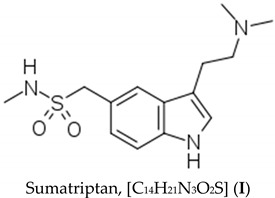


*S. aureus* produces coagulase and hyaluronidase. *B. subtilis* produces a variety of extracellular enzymes, including proteases, α-amylase, a levansucrase, β-glucanases and different lipolytic enzymes [7,8,10]. *P. aeruginosa* produces proteases, including elastase, deoxyribonuclease and lactamase, with broader substrate specificities. *Salmonella* spp. encode four superoxide dismutases (SodA, SodB, SodCI, SodCII), three catalases (KatE, KatG, KatN) and three peroxiredoxins (AhpC, TsaA, Tpx) [11,12,13]. These organisms were selected due to their importance in various biotransformations and also because they are commonly known for their biotransformation capabilities [14,15,16]. The objective of the present work was to study the biotransformation of sumatriptan by *S. aureus*, *B. subtilis*, *P. aeruginosa* and *Salmonella* spp. and to identify the drug metabolites by liquid chromatography–mass spectrometry and nuclear magnetic resonance.

## 2. Results

**Figure 1:**
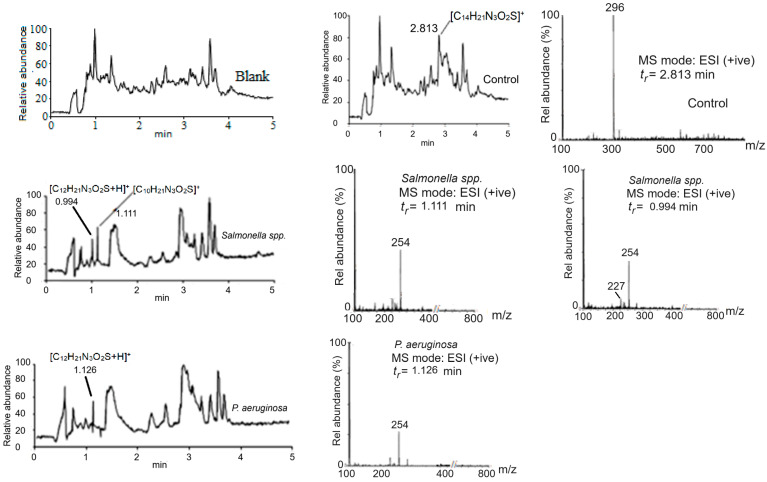

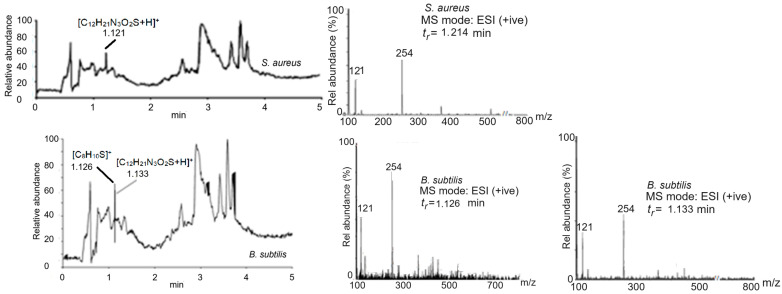
Total ion chromatograms and positive-ion electrospray mass spectra of filtered broth of blank, control, *Salmonella* spp. (ATCC^®^ 14028^TM^), *P. aeruginosa* (ATCC^®^ 9027^TM^), *S. aureus* (ATCC^®^ 6538^TM^) and *B. subtilis* (ATCC^®^ 6633^TM^) after incubation at 37 °C for 14 days.

The total ion chromatograms (TICs) and the respective mass spectra obtained by positive-ion electrospray mass spectrometry are depicted in Figure 1. The void volume of the column was noted to be 0.24 mL. A careful viewing of the TICs revealed the appearance of new peaks at 0.994 min (*Salmonella* spp.), 1.111 min (*Salmonella* spp.), 1.126 min (*B. subtilis*, *P. aeruginosa*), 1.333 min (*B. subtilis*) and 1.214 min (*S. aureus*). These peaks were also observed in the chromatograms obtained by the PDA detector that confirmed the correct working of the system. The fragmentation patterns of these metabolites are listed in Table 1. We attempted to calculate some of the important properties with well-known computational software (T.E.S.T Version 5.1.2 and SwissADME); these are listed in Table 2.

## 3. Discussion

**Scheme 1:**
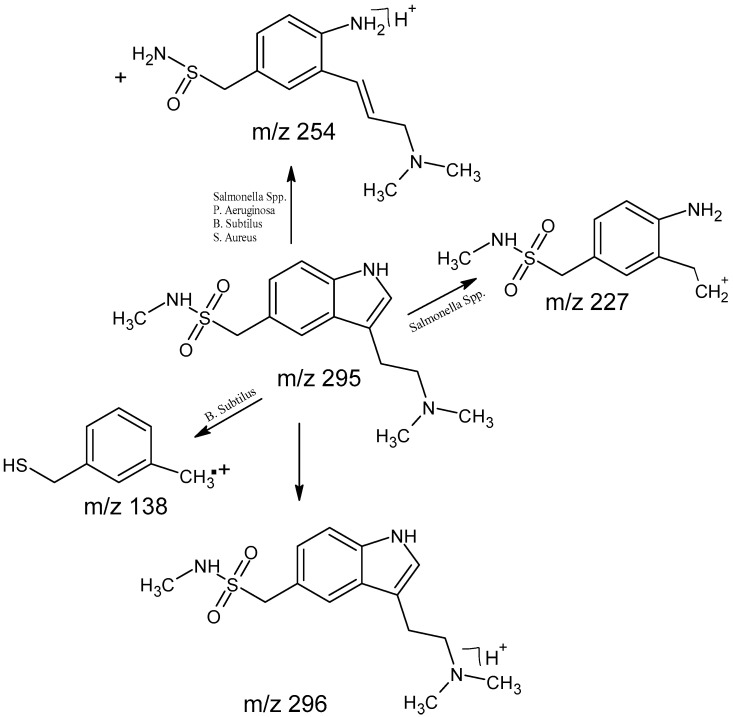
Metabolites of sumatriptan.

The results of this study demonstrated that the drug sumatriptan is metabolized by *S. aureus*, *B. subtilis*, *P. aeruginosa* and *Salmonella* spp. The metabolites were successfully separated by a C18 Acquity ultra-performance liquid chromatography (UPLC) column and identified by mass spectroscopy and nuclear magnetic resonance (NMR) analysis. The mass spectra of the corresponding peaks at specific retention times suggested the presence of (3-methylphenyl)methanethiol (*B. subtilis*), 1-(4-amino-3-ethylphenyl)-N-methylmethanesulfonamide (*Salmonella* spp.) and 1-{4-amino-3-[(1E)-3-(dimethylamino)prop-1-en-1-yl]phenyl}methanesulfinamide (*S. aureus*, *B. subtilis*, *P. aeruginosa*, *Salmonella* spp.) as the metabolites produced according to Scheme 1. The identity of (3-methylphenyl)methanethiol was validated by the retention time produced by its standard.

The metabolite (3-methylphenyl)methanethiol (*m*/*z* 138) appears to be produced by *B. subtilis* as a result of demethylation and oxidative deamination of the parent molecule, whereas the 1-(4-amino-3-ethylphenyl)-N-methylmethanesulfonamide (*m*/*z* 227) produced by *Salmonella* spp. is the result of oxidative deamination. On the other hand, the 1-{4-amino-3-[(1E)-3-(dimethylamino)prop-1-en-1-yl]phenyl}methanesulfinamide (*m/z* 254) produced by all four organisms is the result of demethylation at the methanesulfonamide group and oxidative destruction of the indole moiety in the molecule. These products were also characterized by ^1^HNMR and ^13^CNMR analyses (Section 4.4). As these metabolites are produced as a result of demethylation and/or oxidative deamination, it can be hypothesized that sumatriptan will be metabolized in the liver and central nervous system where such transformations can occur, as reported for MAO A [17]. *B. subtilis* is commonly found in the bowel as part of the normal flora, *Salmonella* spp. is present during active infections, and *S. aureus* and *P. aeruginosa* are not typically found in the bowel under normal conditions but can be present in specific pathological situations, so the metabolization of sumatriptan by these organisms can occur in the bowel. As far as metabolization in the liver or central nervous system is concerned, it will be mediated independently by the monoamine oxidase A present there; this has no association with the bacteria, as they do not produce this enzyme. Keeping these results in mind, parenteral or nasal delivery can be suggested as preferred routes of administration to reduce first-pass metabolism. The benefits of nasal delivery include the large surface area, porous endothelial membrane, high total blood flow, avoidance of first-pass metabolism and ready accessibility.

It was noticed that all four organisms used in this work attacked the indole moiety in sumatriptan; this information is important to address the resistance problem associated with the drug. In light of the findings of this study, it may be postulated that sumatriptan resistance may develop due to the prevalence of infections caused by the organisms under investigation. The metabolite (3-methylphenyl)methanethiol is a known chemical (CAS # 25697-56-7) that is mainly used in proteomics research applications; however, the other two are new, and their pharmacological properties are not known. Comparing the median lethal dose (LD_50_) values of these metabolites (201–619 mg kg^−1^) with that of oral sumatriptan succinate (2939 mg kg^−1^), they appear to be highly toxic. One of them, (3-methylphenyl)methanethiol, can cross the blood–brain barrier (BBB) and is a cytochrome P450 1A2 inhibitor. All three exhibit high gastrointestinal absorption. These parameters are indicative of higher toxicity that may be caused by sumatriptan, especially to the central nervous system, where the drug is expected to be metabolized.

## 4. Materials and Methods

### 4.1. Materials

The chemicals and solvents used were sumatriptan succinate (NCPC Hebei Huamin Pharmaceutical Co., Ltd., Shijiazhuang City, Hebei Province, China); (3-methylphenyl)methanethiol, used as a standard (Sigma-Aldrich, San Jose, CA 95134, USA); ammonium acetate (Sigma-Aldrich, USA); methanol (Honeywell, Charlotte, NC, USA); acetonitrile (E. Merck, Darmstadt, Germany). *S. aureus* (ATCC^®^ 6538^TM^), *B. subtilis* (ATCC^®^ 6633^TM^), *P. aeruginosa* (ATCC^®^ 9027^TM^), *Salmonella* spp. (ATCC^®^ 14028^TM^) and tryptic soy broth (TSB) were also used (Sigma-Aldrich, San Jose, CA 95134, USA). All of the solvents were of MS analytical grade and used as received.

### 4.2. Biotransformation Study

Blank preparation: The TSB (0.5 mL) was treated with acetonitrile (1 mL) and centrifuged (*@* 3000 rpm) for 5 min. To the supernatant (0.5 mL), water (0.5 mL) was added, and the solution was filtered through a 0.45 µm membrane filter (labelled S1).

Control preparation: Sumatriptan succinate (100 mg) was dissolved in TSB to make 100 mL; the mixture was incubated at 37 °C for 14 days. After this time, the broth (0.5 mL) was treated with acetonitrile (1 mL), shaken and centrifuged (@ 3000 rpm) for 5 min. To the supernatant (0.5 mL), water (0.5 mL) was added, and the mixture was filtered through a 0.45 µm membrane filter to obtain S2.

Growth cultures: The cells were inoculated in TSB (100 mL) and incubated in a shaking incubator at 30 °C until the turbidity was >0.5 MacFarland standard (prepared by adding 0.5 mL of 0.048 M BaCl_2_ to 99.5 mL of 0.18 M H_2_SO_4_). This was labelled S3.

Biotransformation: Sumatriptan succinate (100 mg) and S3 (1 mL) were transferred to a 100 mL flask. To this, TSB was added to make 100 mL. The mixture was transferred to a culture bottle and incubated at 37 °C for 14 days. After this time, the broth (0.5 mL) was treated with acetonitrile (1 mL), shaken and centrifuged (*@* 3000 rpm) for 5 min. To the supernatant (0.5 mL), water (0.5 mL) was added, and the mixture was filtered through a 0.45 µm membrane filter to obtain S4.

### 4.3. High-Performance Liquid Chromatography–Photometric Diode Array (HPLC-PDA) and Liquid Chromatography–Mass Spectrometric (LC-MS) Analyses

Preparation of mobile phase: A 500 mL mixture of methanol–acetonitrile (100:400) and a buffer of 5 mM ammonium acetate in water were prepared. The mobile phase used was 10% methanol–acetonitrile mixture plus 90% buffer after filtration through a 0.45 µm membrane filter.

Preparation of standard: An accurately weighed amount of sumatriptan succinate (100 mg) was dissolved in water to make 100 mL and filtered through a 0.45 µm membrane filter.

Preparation of sample: Acetonitrile (1 mL) was added to the filtered treated broth (0.5 mL); the mixture was shaken and centrifuged (@ 3000 rpm) for 5 min. To the supernatant (0.5 mL), water (0.5 mL) was added, and the solution was filtered through a 0.45 µm membrane filter.

Chromatography: HPLC was performed on a Waters Acquity TQD LC/MS/MS System using a 1.7 μm ACQUITY UPLC BEH C18 column (2.1 × 100 mm; Waters^TM^, Milford, MA 01757, USA) with a PDA detector at 282 nm, a flow rate of 0.4 mL min^−1^, a column temperature of 40 °C and an injection volume of 5 μL. The S1, S2, S4 and standard solutions were chromatographed.

LC-MS: The LC-MS analysis was performed on a Waters Acquity TQD LC/MS/MS System equipped with Xevo^®^ TQD, using the same chromatographic conditions as above. The data were processed by MassLynx software, version 4.1 (Waters^TM^, Milford, MA 01757, USA). The mass spectrometer was operated in positive ESI mode. The other parameters were as follows: collision gas, curtain gas, ion source gas 1 and ion source gas 2 (nitrogen) pressures 6, 0.2, 0.9 and 50 psi, respectively; source voltage 2.8 kV, cone voltage 30 V; desolvation temperature 550 °C; source gas flow 1000 L h^−1^. The spectra were acquired over the mass range 100–800 (*m*/*z*). The fragmentation patterns were analyzed by applying the standard procedures. The S1, S2, S4 and standard solutions were chromatographed.

Collection of biotransformation products: The biotransformation products were collected at the respective retention times using a semipreparative accessory and subjected to NMR analysis.

Computation of pharmacological properties: The pharmacological properties were calculated by SwissADME Methodologies [http://www.swissadme.ch/], accessed on 15 June 2024, and LD50 values were calculated by Toxicity-Estimation-Software-Tool (T.E.S.T) Version 5.1.2 [https://www.epa.gov/chemical-research/toxicity-estimation-software-tool-test]. For these calculations, structures of the subject molecules were drawn in ChemSketch 12 freeware [https://www.acdlabs.com/resources/free-chemistry-software-apps/chemsketch-freeware/] and saved as a ‘*.mol’ file; ‘SMILES’ notation was generated as required by these software tools, and the files were uploaded online. The results were generated instantly.

### 4.4. NMR Analysis

^1^HNMR and ^13^CNMR spectra were recorded in DMSO-d_6_ by a Bruker Avance 400 MHz spectrometer (^1^H, 400.00 MHz and ^13^C 100.00 MHz) with tetramethylsilane as the reference. The chemical shifts (ppm) were as follows: C_8_H_10_S, ^1^HNMR, δ 2.2 (3H, s), 3.8 (2H, s), 6.9–7.1 (2H, 7.0 (ddd, *J* = 7.9, 1.4, 1.2 Hz), 7.1 (ddd, *J* = 7.8, 1.5, 1.3 Hz)), 7.1–7.3 (2H, 7.2 (td, *J* = 1.3, 0.4 Hz), 7.2 (td, *J* = 7.8, 0.5 Hz)); ^13^CNMR, δ 21.2 (1C, s), 28.7 (1C, s), 127.4 (1C, s), 127.8 (1C, s), 128.0 (1C, s), 128.6 (1C, s), 134.8 (1C, s), 136.0 (1C, s). C_10_H_16_N_2_O_2_S, ^1^HNMR, δ 1.0 (3H, t, *J* = 7.5 Hz), 2.6 (2H, q, *J* = 7.4 Hz), 2.9 (3H, s), 6.4 (1H, dd, *J* = 7.6, 0.5 Hz), 6.7 (1H, dd, *J* = 7.6, 1.5 Hz), 6.9 (1H, dd, *J* = 1.5, 0.4 Hz); ^13^CNMR: δ 14.7 (1C, s), 23.8 (1C, s), 29.1 (1C, s),59.0 (1C, s),114.8 (1C, s), 123.4 (1C, s), 126.7 (1C, s), 127.2 (1C, s),127.4 (1c, s),144.1 (1C, s). C_12_H_19_N_3_OS, ^1^HNMR: δ 2.4 (6H, s), 2.8–3.0 (2H, 2.9 (d, *J* = 7.0 Hz), 2.9 (d, *J* = 7.1 Hz)), 4. 9-5.0 (2H, 4.9 (s), 4.9 (s)), 6.2 (1H, dt, *J* = 17.2, 7.0 Hz), 6.4-6.5 (2H, 6.4 (d, *J* = 17.1 Hz), 6.4 (dd, *J* = 7.5, 0.5 Hz)), 6.8 (1H, dd, *J* = 7.7, 1.9 Hz), 7.1 (1H, dd, *J* = 1.7, 0.5Hz); ^13^CNMR: δ 33.7 (1C, s),45.0 (2C, s),49.2 (1C, s),114.6 (1C, s), 123.2 (1C, s),123.5 (1C, s), 127.2 (1C, s), 127.5 (1C, s), 128.3 (1C, s), 136.0 (1C, s), 145.5 (1C, s).

## 5. Conclusions

Sumatriptan is metabolized by *S. aureus*, *B. subtilis*, *P. aeruginosa* and *Salmonella* spp., producing (3-methylphenyl)methanethiol (*B. subtilis*), 1-(4-amino-3-ethylphenyl)-N-methylmethanesulfonamide (*Salmonella* spp.) and 1-{4-amino-3-[(1E)-3-(dimethylamino)prop-1-en-1-yl]phenyl}methanesulfinamide (*Salmonella* spp., *B. subtilis*, *P. aeruginosa*, *S. aureus*) as identified by LC-MS analysis. The metabolite (3-methylphenyl)methanethiol is a known chemical (CAS # 25697-56-7), while the other two are new. (3-Methylphenyl)methanethiol can cross the BBB and is a cytochrome P450 1A2 inhibitor. The calculated LD_50_ values of these metabolites (201–619 mg kg^−1^) indicate that they are much more toxic than sumatriptan succinate (2939 mg kg^−1^). These properties were calculated, not determined experimentally, which is a limitation of this study. We intend to test these compounds in vivo.

## Figures and Tables

**Table 1:** Mass-spectral fragmentation patterns of metabolites of sumatriptan produced by various organisms.

Organism	TIC, t_r_ (min)	[M]^+^ (amu)	Fragmentation Pattern (amu)
*B. subtilis*	1.126	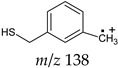 [C_8_H_10_S]^+^	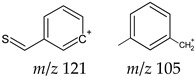
*Salmonella* spp.	1.111	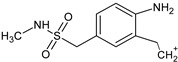 [C_10_H_21_N_3_O_2_S]^+^	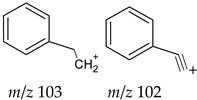
*Salmonella* spp.	0.994	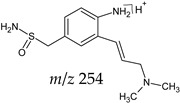 [C_12_H_21_N_3_O_2_S+H]^+^	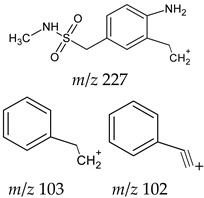
*P. aeruginosa*	1.126		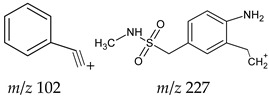
*B. subtilis*	1.133		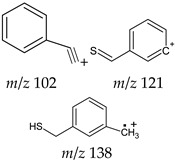
*S. aureus*	1.214		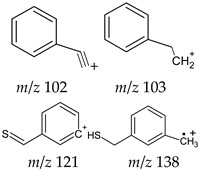

**Table 2:** Pharmacological properties of the metabolites of sumatriptan produced by *B. subtilis*, *Salmonella* spp., *P. aeruginosa* and *S. aureus*.

Metabolite	LD_50_ (rat) ^a^ mg kg^−1^	Pharmacological Properties ^b^
(3-methylphenyl)methanethiol (M_w_ 138.23 g mol^−1^; CAS # 25697-56-7)	618.96	Gastrointestinal absorption: High BBB permeation: Yes Cytochrome P450 1A2 inhibitor: Yes—could result in increased toxicity for CYP1A2 substrates CYP2C19 inhibitor: No, CYP2C9 inhibitor: No CYP2D6 inhibitor: No, CYP3A4 inhibitor: No
1-(4-amino-3-ethylphenyl)-N-methylmethanesulfonamide (M_w_ 228.31 g mol^−1^)	2461.97	Gastrointestinal absorption: High BBB permeation: No Cytochrome P450 1A2 inhibitor: No CYP2C19 inhibitor: No, CYP2C9 inhibitor: No CYP2D6 inhibitor: No, CYP3A4 inhibitor: No
1-{4-amino-3-[(1E)-3-(dimethylamino)prop-1-en-1-yl]phenyl}methanesulfinamide (M_w_ 253.36 g mol^−1^) Sumatriptan succinate	201 2939	Gastrointestinal absorption: High BBB permeation: No Cytochrome P450 1A2 inhibitor: No CYP2C19 inhibitor: No, CYP2C9 inhibitor: No CYP2D6 inhibitor: No, CYP3A4 inhibitor: No

^a^ Calculated by use of T.E.S.T software Version 5.1.2 [https://www.epa.gov/chemical-research/toxicity-estimation-software-tool-test], accessed on 15 June 2024. ^b^ Calculated by use of SwissADME Methodologies [http://www.swissadme.ch/], accessed on 15 June 2024. Abbreviations: LD_50_—median lethal dose; M_w_—molecular weight; BBB—blood–brain barrier; CYP—cytochrome P enzymes.

## Data Availability

Data are contained within the article.
